# Beneficial Changes in Growth Performance, Antioxidant Capacity, Immune Response, Hepatic Health, and Flesh Quality of *Trachinotus ovatus* Fed With *Oedocladium carolinianum*

**DOI:** 10.3389/fimmu.2022.940929

**Published:** 2022-07-04

**Authors:** Wei Zhao, Xin Cui, Zi-Qiao Wang, Rong Yao, Shi-Hua Xie, Bao-Yan Gao, Cheng-Wu Zhang, Jin Niu

**Affiliations:** ^1^ State key Laboratory of Biocontrol, Guangdong Provincial Key Laboratory for Aquatic Economic Animals and Southern Marine Science and Engineering Guangdong Laboratory (Zhuhai), School of Life Sciences, Sun Yat-Sen University, Guangzhou, China; ^2^ Department of Ecology, Institute of Hydrobiology, College of Life Science and Technology, Jinan University, Guangzhou, China

**Keywords:** *Trachinotus ovatus*, *Oedocladium carolinanum*, antioxidant status, growth performance, immunity, histomorphology

## Abstract

The purpose of this study is to assess the feasibility of astaxanthin-rich *Oedocladium carolinianum* as an immunostimulant in the diet for *Trachinotus ovatus*. Three experimental diets containing 0% (OC0), 1% (OC1), and 5% (OC5) *O*. *carolinianum* powder were formulated for 6-week feeding trials. The results indicated that the OC5 diet boosted the growth performance through decreasing the feed conversion ratio and increasing digestive enzyme activities and intestinal villus length. Meanwhile, fish fed with the OC5 diet promoted antioxidant ability *via* stimulating the Nrf2-ARE signal pathway and enhancing antioxidant enzyme activities. Furthermore, the OC5 diet exerted hepatoprotective effects by suppressing the lipid deposition and inflammation response and enhancing the transport capacity of cholesterol. Besides, the OC5 diet improved the non-specific immunity by activating the lysozyme and complement system and increasing the nitric oxide content and total nitric oxide synthase activity. Dietary *O*. *carolinianum* supplementation promoted the deposition of astaxanthin in the whole body. Therefore, a diet supplemented with 5% *O*. *carolinianum* is recommended to boost the growth, antioxidant capacity, immune response, and flesh quality of *T. ovatus*.

## Introduction

Astaxanthin, a xanthophyll carotenoid with superior antioxidative property, has been found in aquatic animals (lobster, crab, shrimp, fish), yeast *Phaffia rhodozyma*, bacterium *Paracoccus carotinifaciens*, and some microalgae (*Haematococcus pluvialis*, *Chromochloris zofingiensis* (former name: *Chlorella zofingiensis*), *Scenedesmus obliquus*) (1, 2). The antioxidant activity of astaxanthin was significantly stronger than that of zeaxanthin, lutein, lycopene, canthaxanthin, β-carotene, and α-tocopherol (3). Astaxanthin with its anti-inflammatory, antioxidative, antidiabetic, anticancer, antiaging, and immunomodulation effects has been widely used in the healthcare products, foods, feeds, pharmaceuticals, and cosmetics (4, 5). Over the past decade, astaxanthin has also attracted great interest as a novel aquafeed additive. Dietary astaxanthin or astaxanthin-rich microalgae supplementation had positive effects on weight gain, antioxidant capacity, pigmentation, stress resistance, and immune regulation of aquatic animals (6–9). The application of astaxanthin in aquatic feed is considered to be a feasible pathway for the sustainable development of aquaculture, since it can reduce the use of antibiotics.

Up to now, commercial astaxanthin mainly comes from *P. rhodozyma*, *H. pluvialis*, and chemical synthesis. Synthetic astaxanthin dominates the market with its cost-effective advantages, covering 95% of the global astaxanthin market (10). However, astaxanthin from *H. pluvialis* is 50 times and 20 times stronger than synthetic astaxanthin in singlet oxygen quenching and free radical elimination (11). Besides, Su etal. (12) suggested that *H. pluvialis* powder could be better to improve the astaxanthin accumulation and nutritive quality of *Eriocheir sinensis* than synthetic astaxanthin. Similarly, dietary *H. pluvialis* supplementation can improve the growth and antioxidant capacity of *Pseudosciaena crocea* more than synthetic astaxanthin (9). The different biological efficacy of synthetic astaxanthin and astaxanthin from *H. pluvialis* is mainly due to the difference in existing form and molecular structure. Firstly, astaxanthin from *H. pluvialis* is a mostly esterified form while synthetic astaxanthin is an unesterified form (5). The stability and bioavailability of esterified astaxanthin are better than those of unesterified astaxanthin. Secondly, the primary stereoisomer of astaxanthin from *H. pluvialis* is (3S,3′S), whereas synthetic astaxanthin comprises three stereoisomers, namely, (3S,3′S), (3R,3′S), and (3R,3′R), in a ratio of 1:2:1 (11, 12). Astaxanthin in the form of (3S,3′S) showed higher antioxidant and antiaging activities than that in the form of (3R,3′R) and (3R,3′S) *in vivo* and *in vitro* (13). Therefore, the dosage level for synthetic astaxanthin is greater than that for astaxanthin from *H. pluvialis* in order to achieve a similar antioxidant activity. In general, as an aquafeed additive, astaxanthin from *H. pluvialis* has advantages over synthetic astaxanthin. In practical application, it is a better choice to use astaxanthin-rich *H. pluvialis* powder as an aquafeed additive due to the high cost of astaxanthin extraction from *H. pluvialis*. However, as a unicellular microalga, *H. pluvialis* is susceptible to microbial contamination during the cultivation process, resulting in a reduction in biomass yield or even a complete loss of biomass yield (14). Additionally, harvesting and dewatering of the *H. pluvialis* cells is expensive and requires the use of centrifuges or plate-and-frame filter press (4). Therefore, this largely limits the application scale of *H. pluvialis* powder in aquafeed due to the high production cost and sales price.

*Oedocladium carolinianum* is a filamentous green microalga that can produce esterified astaxanthin under stress conditions (4, 15). We previously reported that *O. carolinianum* can produce 1.62% (dry weight) astaxanthin under stress conditions (16). Wang etal. (4) suggested that *O. carolinianum* produced up to 3.91% (dry weight) astaxanthin under nitrogen starvation and salinity stress. More importantly, filamentous microalgae can be harvested by gravity sedimentation and filtration and are not easily preyed by protozoa and bacterial contamination during the cultivation process. These advantages of filamentous microalgae during cultivation and harvesting are important for reducing the production cost of microalgae. Therefore, *O. carolinianum* is a promising microalga for a sustainable large-scale production of astaxanthin. Previous studies reported that dietary *O. carolinianum* supplementation significantly improved the antioxidant capacity and flesh quality of *Carassius auratus gibelio* (15). Therefore, *O. carolinianum* has the potential to replace *H. pluvialis* as another important source of astaxanthin in aquafeed.

Golden pompano (*Trachinotus ovatus*) is a commercially important fish widely distributed in tropical and subtropical areas, such as China, Japan, Australia, and Southeast Asia (17). In recent years, sea cage culture of *T. ovatus* has been getting hotter in southern China, Malaysia, and Singapore because of its fast growth, delicious meat, high nutrition value, and increasing market demand. However, owing to intensive aquaculture, water environment pollution, and extreme weather, the immunity and disease resistance of *T. ovatus* cultured in sea cages are declining and thus are more susceptible to various environmental stressors and infectious diseases. The chemical drugs used against such infectious diseases have not produced satisfactory effects, and even threatened food safety. Therefore, dietary immunostimulant supplementation to increase the immunity and disease resistance is the key strategy for the successful cultivation of *T. ovatus*.

The present study evaluated the feasibility of astaxanthin-rich *O. carolinianum* powder as an immunostimulant in the diet for *T. ovatus*. Accordingly, a nutritional feeding test was performed to assess the effects of dietary *O. carolinianum* powder on the growth, antioxidant status, immune response, hepatic health, and flesh quality of *T. ovatus*. The results of the study can enrich the types of application of microalgae and the source of astaxanthin in aquafeed.

## Materials and Methods

### Microalgae Culture and Diet Preparation

**Figure 1:**
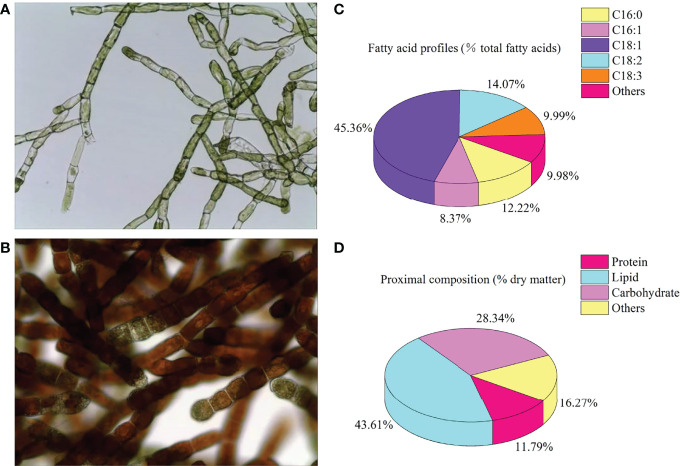
Cell morphology **(A)**, green cell stage; **(B)**, red cell stage), fatty acid profiles **(C)**, % total fatty acids), and proximal composition **(D)**, % dry matter) of *Oedocladium carolinianum*.

The stock culture of *O. carolinianum* was carried out in a flat glass photobioreactor (light path: 6 cm; length: 240 cm; height: 120 cm) with a two-step batch culture strategy. In the stage of green cell vegetative culture, *O. carolinianum* cultures were grown in mBBM medium (18) containing 9.0 mM NaNO_3_ for 12 days. The flat glass photobioreactor was maintained at 25°C with continuous unilateral lighting at 100 μmol photons m^-2^ s^-1^ and aerated with 1.0% CO_2_ (v/v). Then, *O. carolinianum* was transferred into nitrogen-free mBBM medium for 12-day astaxanthin accumulation cultivation (red cell stage). Continuous bilateral illumination of 300 μmol photons m^-2^ s^-1^ was maintained during the red cell stage. Other culture conditions were consistent with the green cell stage. At the end of culture, microalgal cells were harvested by gauze filtration and then freeze-dried by a freezing dryer to obtain microalgal powder. The *O. carolinianum* powder contained 0.62% astaxanthin, 5.33% linoleic acid, and 3.79% linolenic acid (**Figure 1**).

Three isonitrogenous and isolipidic diets containing 0% (OC0), 1% (OC1), and 5% (OC5) *O. carolinianum* were formulated. The formula and proximate composition analysis of experimental diets is shown in **Table 1**. According to the procedure described by Zhao etal. (17), experimental diets were manufactured into 1.2-mm-diameter puffed pellets and stored at -20°C.

**Table 1:** Composition and nutrient levels of the experimental diets (% dry matter).

Ingredients	OC0	OC1	OC5
Fish meal^1^	45	45	45
Soybean meal^1^	16.3	16	15
Wheat flour^1^	20	20	20
Beer yeast^1^	3	3	3
Microcrystalline cellulose	4	3.7	2.4
Fish oil^1^	7	6.6	4.9
Soybean lecithin^1^	1	1	1
Ca(H_2_PO_4_)_2_^1^	1	1	1
Vitamin premix^a^	1	1	1
Mineral premix^b^	1	1	1
Choline^1^	0.5	0.5	0.5
Vitamin C^1^	0.2	0.2	0.2
*Oedocladium carolinianum*	0	1	5
Total	100	100	100
Nutrient levels^c^ (% dry matter)			
Crude lipid	12.47	12.31	11.88
Crude protein	42.02	42.27	42.56
Moisture	9.01	9.05	8.73
Ash	10.21	10.14	10.62
Astaxanthin (g kg^-1^ dry matter)	0	0.06	0.31

^1^Obtained from Kyorin Industry (Shenzhen) Co., Ltd., Wuxi, China. Fish meal, crude protein 65.0%, crude lipid 8.9%; soybean meal, crude protein 48.5%, crude lipid 1.5%; wheat flour, crude protein 13.8%, crude lipid 2.8%; beer yeast, crude protein 48.0%, crude lipid 3.9%.

a Vitamin premix provides the following per kg of diet: VB_1_ 25 mg, VB_2_ 45 mg, pyridoxine HCl 20 mg, VB_12_ 0.1 mg, VK_3_ 10 mg, inositol 800 mg, pantothenic acid 60 mg, niacin acid 200 mg, folic acid 20 mg, biotin 1.20 mg, retinal acetate 32 mg, cholecalciferol 5 mg, α-tocopherolα 120 mg, ascorbic acid 2000 mg, choline chloride 2500 mg, ethoxyquin 150 mg, wheat middling 14.012 g.

b Mineral premix provides the following per kg of diet: NaF 2 mg, KI 0.8 mg, CoCl_2_·6H_2_O 50 mg, CuSO_4_·5H_2_O 10 mg, FeSO_4_·H_2_O 80 mg, ZnSO_4_·H_2_O 50 mg, MnSO_4_·H_2_O 60 mg, MgSO_4_·7H_2_O 1200 mg, Ca(H_2_PO_4_)_2_·H_2_O 3,000 mg, NaCl 100 mg, zeolite 15.447 g.

c Measured values.

### Fish Management

Juvenile *T. ovatus* were obtained from a commercial company in Lingshui, Hainan, China. The feeding trial was conducted at Lingshui bay (Lingshui, Hainan, China). Before the feeding trail, *T. ovatus* juveniles were acclimated to the laboratory conditions and fed two times daily with a control diet for 14 days. Then, 180 fish were selected (initial body weight 7.05 ± 0.12 g) and randomly assigned into nine sea cages (1.5 m × 1.5 m × 2.0 m, three cages per diet) at a density of 20 fish per cage. Fish were fed slowly by hand to apparent satiation two times a day (08:30 and 17:30) for 42 days. Feed consumption and the number and weight of dead fish were recorded every day. During the feeding trial, the water temperature and salinity ranged from 28°C to 31°C and from 30 to 32 g/l, respectively. The dissolved oxygen content was above 6 mg/l, and fish were cultured under a natural light–dark cycle.

### Sampling Collection

The animal use protocol listed below has been reviewed and approved by the Institution Animal Care and Use Committee, Sun Yat-Sen University. At the end of the feeding trial, all fish were fasted for 24 h and then anesthetized with 20 mg l^-1^ of tricaine methanesulfonate (Sigma, St. Louis, MO, USA). All fish in each cage were counted and individually weighed to evaluate growth performance. Blood, from six fish per cage, were collected from the caudal vein. Blood samples were stored overnight at 4°C and centrifuged (4°C, 4,000 r/min, 10 min) to collect the serum. Then, serum samples were stored at -80°C for analysis of hematological parameters and antioxidant enzyme activities. The liver and midgut of the aforementioned six fish were rapidly removed and frozen in liquid nitrogen and then stored at -80°C for analysis of digestive enzyme, antioxidant enzyme, and gene expression, respectively. Besides, three fish in each cage were collected and frozen in liquid nitrogen and then conserved at -80°C for analysis of fatty acid composition and astaxanthin content of the whole body. Finally, the liver and midgut samples, from three fish per cage, were removed and fixed in 4% paraformaldehyde for histological analysis.

### Biochemical Analysis of Microalgae, Experimental Diets, and Whole Body

The total lipids in the *O. carolinianum* powder were measured with the gravimetric method following the procedure described by Gao etal. (19). The fatty acid profiles of *O. carolinianum* powder were determined using a gas chromatograph (6890 N GC, Agilent Technologies, USA) according to the protocol designed by Zhang etal. (20). The total carbohydrate of *O. carolinianum* powder was quantified with the phenol-sulfuric acid method following the procedure of Wang etal. (21). The total protein of *O. carolinianum* powder was determined by the Lowry method using commercial assay kits (Sangon Biotech, Shanghai, China).

Crude protein, crude lipid, moisture and ash contents in experimental diets were measured following the method described by Zhao etal. (22).

The astaxanthin contents of *O. carolinianum* powder, experimental diets, and whole body were measured by spectrophotometry using the procedure described by Wang et al. (23).

The fatty acid profiles of whole body were quantified using a gas chromatograph (6890 N GC, Agilent Technologies, USA) according to the method described by (24).

### Histological Observation

Midgut and liver samples were fixed in 4% paraformaldehyde and then dehydrated in a graded ethanol series (75%, 4 h; 85%, 2 h; 90%, 2 h; 95%, 1 h; 100%, 1 h) and embedded in paraffin. Sections (5 μm thick) of the midgut and liver were obtained with a rotary microtome and stained with hematoxylin and eosin. Finally, the sections were observed and photographed using an optical microscope (Leica DMLB, Germany).

### Enzyme Activity Assays

The midgut and liver samples were homogenized in ice-cold normal saline (1:10 dilution) and centrifuged at 3,000 r/min (4°C) for 20 min to obtain the supernatant. The enzyme activities in the supernatant and serum were determined by utilizing commercial kits (Nanjing Jiancheng Bioengineering Institute, Nanjing, China) based on the manufacturer’s instructions. The absorbance value was detected by microplate spectrophotometer Epoch (BioTek, Winooski, USA).

The activities of superoxide dismutase (SOD), glutathione peroxidase (GSH-PX), catalase (CAT), malondialdehyde (MDA) content, and total antioxidant capacity (T-AOC) in the liver were detected by the relevant kits (Cat. No. A001-1, A005, A007-1, A003-1, and A015-2-1, respectively).

The activities of superoxide dismutase (SOD) and glutathione peroxidase (GSH-PX), and malondialdehyde (MDA) content in the serum were measured by the relevant kits (Cat. No. A001-1, A005, and A003-1, respectively).

The activities of amylase (AMS), pepsin (PEP), and lipase (LPS) in the midgut were determined by the relevant kits (Cat. No. C016, A080-1-1, and A054-2, respectively).

### Immune-Related Parameter Assays

Immune-related parameters in the liver and serum were assayed using commercial kits (Nanjing Jiancheng Bioengineering Institute, Nanjing, China) following the provided instructions, including nitric oxide (NO) content and total nitric oxide synthase (TNOS) activity in the liver, and lysozyme activity and complement 4 (C4) in serum.

### Serum Parameter Assays

Automatic biochemical analyzer Chemray 240 (Rayto Life Science Co., Ltd., Shenzhen, China) and corresponding commercial kits (Huili Biotech Co., Ltd., Changchun, China) were used to determine the contents of triglyceride (TG), glucose (GLU), low-density lipoprotein cholesterol (LDL-C), and high-density lipoprotein cholesterol (HDL-C), as well as the activities of aspartate aminotransferase (AST) and alanine aminotransferase (ALT) in the serum.

### RNA Extraction and Gene Expression Analysis

The total RNA extraction and subsequent quantitative reverse transcription polymerase chain reaction (qRT-PCR) were performed according to the procedures described by Zhao etal. (17). Briefly, the total RNA of the liver in each cage was extracted using a reagent kit (TaKaRa, Dalian, China). Agarose gel electrophoresis at 1% and spectrophotometric analysis (OD260/280) were used to assess the quality and quantity of RNA. Then, the total RNA samples were diluted to the same concentration with diethylpyrocarbonate-treated water for normalization. Subsequently, cDNA was synthesized using a PrimeScript RT Reagent Kit with gDNA Eraser (TaKaRa, Dalian, China) following the manufacturer’s instructions. qRT-PCR for the target genes was carried out using a LightCycler 480 Real-Time System (Roche Applied Science, Basel, Switzerland) with SYBR^®^ Premix ExTaq™ II (TaKaRa, Dalian, China). β-Actin was set as the housekeeping gene. All the primers for qRT-PCR were consistent with our previous study (17); primer sequences are presented in **Supplementary Table 1**. The relative expression levels of target genes were calculated based on the 2^-ΔΔCT^ method (25).

### Statistical Analysis

The specific growth ratio (SGR), survival rate (SR), weight gain rate (WGR), and feed conversion ratio (FCR) were calculated according to the equation described by Zhao etal. (22).

The results were presented as the means ± standard error (SE) and analyzed using SPSS 20.0 statistical software (SPSS, Chicago, IL, USA). All data were checked for normality and homogeneity using the Kolmogorov–Smirnov test and Levene’s test, respectively. The differences in data were analyzed by using one-way analysis of variance (ANOVA) followed by Tukey test. *P* < 0.05 was considered to be statistically significant.

## Results

### Biological Performance

**Figure 2:**
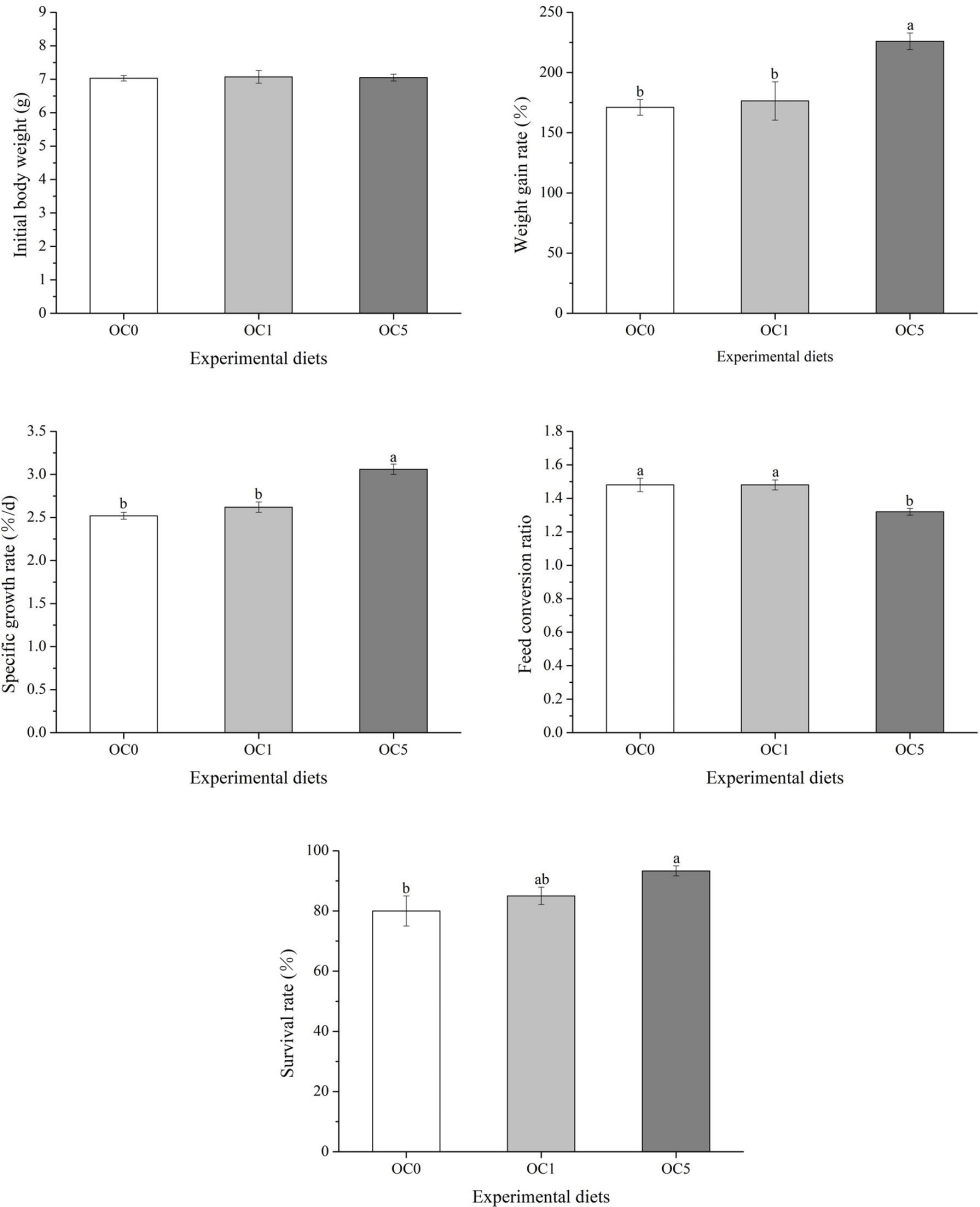
Effects of dietary *Oedocladium carolinianum* powder supplementation on growth performance and feed utilization of *T. ovatus* after the 42-day feeding trial.

The growth performance and feed utilization of *T. ovatus* fed with experimental diets are shown in **Figure 2**. The final body weight (FBW), WGR, and SGR of fish fed with the OC5 diet were significantly higher than those of fish fed with OC0 and OC1 diets (*P* < 0.05). Conversely, the FCR of fish fed with the OC5 diet was significantly less than that of fish fed the OC0 and OC1 diets (*P* < 0.05). Besides, fish fed the OC5 diet showed the highest value of SR, which was significantly higher than that of fish fed the OC0 diet (*P* < 0.05).

### Fatty Acid Composition and Astaxanthin Content of Whole Body

Fatty acid composition and astaxanthin content in the whole body were assayed, as shown in **Table 2**. The total monounsaturated fatty acid (MUFA) concentration was the highest in fish fed the OC5 diet and was significantly higher than that of fish fed the OC0 diet (*P* < 0.05). With regard to MUFAs, dietary *O.carolinianum* powder supplementation significantly increased the oleic acid (C18:1) and eicosenoic acid (C20:1) content (*P* < 0.05), while the lowest palmitoleic acid content was observed in fish fed the OC5 diet (*P* < 0.05). Conversely, the total polyunsaturated fatty acid (PUFAs) and total n‐6 PUFA profiles were the lowest in fish fed the OC5 diet and was significantly lower than that of fish fed the OC0 and OC1 diets (*P* < 0.05). With regard to PUFAs, fish fed the OC5 diet exhibited a lower linoleic acid (C18:2n6) content and a significantly lower one than that of fish fed the OC0 and OC1 diets (*P* < 0.05). Dietary *O. carolinianum* powder supplementation did not affect the total saturated fatty acid (SFAs) and total n‐3 PUFA profiles and n‐3/n‐6 ratio (*P* > 0.05). Among all diet treatments, no significantly differences were observed in the whole-body docosahexaenoic acid (EPA) and docosahexaenoic acid (DHA) concentrations (*P* > 0.05).

**Table 2:** Fatty acid composition (% total fatty acids) and astaxanthin content (mg/kg dry weight) in the whole body of *Trachinotus ovatus* fed with experimental diets.

	OC0	OC1	OC5
Fatty acid composition (% total fatty acids)			
C14:0	8.52 ± 0.17^a^	8.13 ± 0.22^ab^	7.56 ± 0.12^b^
C15:0	0.80 ± 0.01	0.84 ± 0.02	0.82 ± 0.02
C16:0	40.73 ± 0.01	40.28 ± 0.22	40.61 ± 0.07
C17:0	0.75 ± 0.01	0.79 ± 0.03	0.78 ± 0.02
C18:0	9.10 ± 0.13	9.39 ± 0.24	9.65 ± 0.10
C20:0	0.60 ± 0.01^a^	0.64 ± 0.01^b^	0.66 ± 0.01^b^
C14:1	0.04 ± 0.00	0.04 ± 0.00	0.04 ± 0.01
C16:1	8.86 ± 0.14^a^	8.52 ± 0.14^a^	7.47 ± 0.06^b^
C18:1	22.3 ± 0.01^a^	22.95 ± 0.02^b^	24.25 ± 0.09^c^
C20:1	1.19 ± 0.01^a^	1.25 ± 0.01^b^	1.36 ± 0.01^c^
C18:2n6	2.19 ± 0.09^a^	2.15 ± 0.10^a^	1.66 ± 0.02^b^
C18:3n6	0.01 ± 0.00	0.01 ± 0.00	0.02 ± 0.01
C20:2n6	0.17 ± 0.02	0.16 ± 0.01	0.17 ± 0.02
C20:3n6	0.02 ± 0.01	0.05 ± 0.02	0.04 ± 0.01
C20:4n6	0.16 ± 0.01	0.16 ± 0.01	0.16 ± 0.01
C22:2n6	0.12 ± 0.02	0.11 ± 0.01	0.12 ± 0.02
C18:3n3	0.04 ± 0.01	0.04 ± 0.01	0.03 ± 0.00
C20:3n3	0.22 ± 0.00	0.20 ± 0.01	0.20 ± 0.01
C20:5n3 (EPA)	0.15 ± 0.01	0.14 ± 0.03	0.11 ± 0.03
C22:6n3 (DHA)	0.58 ± 0.03	0.53 ± 0.04	0.46 ± 0.07
Others	3.50 ± 0.06	3.68 ± 0.04	3.85 ± 0.02
ΣSFAs	60.49 ± 0.01	60.05 ± 0.28	60.05 ± 0.06
ΣMUFAs	32.39 ± 0.15^a^	32.75 ± 0.17^ab^	33.11 ± 0.04^b^
ΣPUFAs	3.63 ± 0.09^a^	3.52 ± 0.16^a^	2.98 ± 0.02^b^
n-6	2.66 ± 0.11^a^	2.62 ± 0.09^a^	2.16 ± 0.02^b^
n-3	0.98 ± 0.02	0.90 ± 0.08	0.83 ± 0.01
n-3/n-6	0.37 ± 0.02	0.35 ± 0.02	0.38 ± 0.01
Astaxanthin content (mg/kg dry weight)			
Astaxanthin	–	0.25 ± 0.02	2.42 ± 0.16

SFAs, saturated fatty acids; MUFAs, monounsaturated fatty acids; PUFAs, polyunsaturated fatty acids. Values were presented as mean ± SE (n = 3). The small letters indicated significant differences at P < 0.05.

Whole-body astaxanthin contents were 0.25 and 2.42 mg/kg (dry weight) in fish fed the OC1 and OC5 diets, respectively. However, whole-body astaxanthin content was not detected in fish fed the OC0 diet.

### Morphology of Liver and Midgut and Activities of Digestive Enzymes in the Midgut

**Figure 3:**
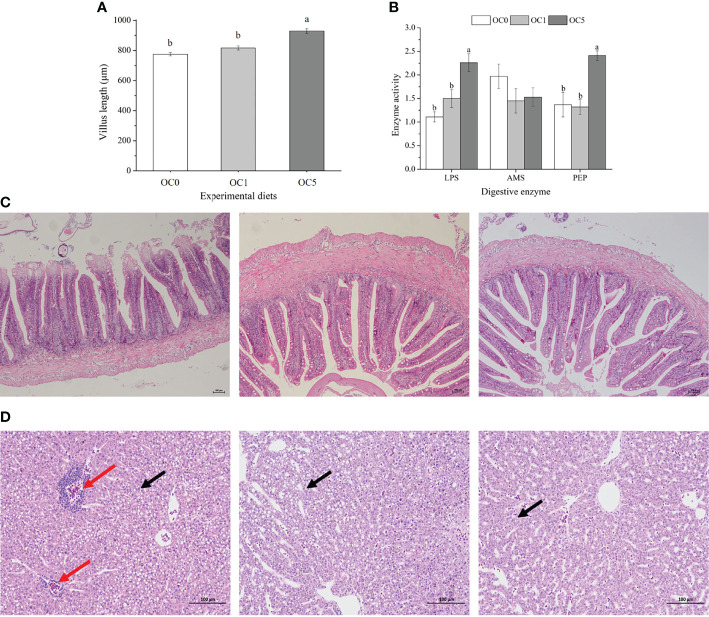
Villus length (**A**, μm) and digestive enzymes activities **(B)** in the mid gut of *T. ovatus* fed experimental diets, and mid-gut **(C)** and liver morphology **(D)** of fish fed OS0 (C-1, D-1), OS1 (C-2, D-2), and OS5 (C-3, D-3) diets. LPS, lipase (U g protein^-1^); AMS, amylase (U mg protein^-1^); PEP, pepsin (U mg protein^-1^). The red arrow indicated the infiltration of inflammatory cells. The black arrow indicated the lipid droplet. Values were presented as mean ± SE (n = 3). The small letters indicated significant differences at *P* < 0.05. Scale bar: 100 μm.

As shown in **Figure 3A**, the villus length of the midgut in fish fed the OC5 diet was significantly higher than that of fish fed the OC0 and OC1 diets (*P* < 0.05). Activities of LPS and PEP in the midgut of fish fed the OC5 diet were significantly higher than those of fish fed the OC0 and OC1 diets (*P* < 0.05) (**Figure 3B**). Besides, AMS activity showed no significant difference among all dietary treatments (*P* > 0.05).

No obvious histological alterations were observed in the midgut among all diet treatments (**Figure 3C**).

The obvious infiltration of inflammatory cells was observed in the liver of fish fed the OC0 diet. However, the liver of fish fed the diet supplemented with *O. carolinianum* showed a healthy morphology. Moreover, the number of lipid droplets in the liver of OC5 diet treatment was less than that of other diet treatments (**Figure 3D**).

### Immune Biochemical Parameters

**Figure 4:**
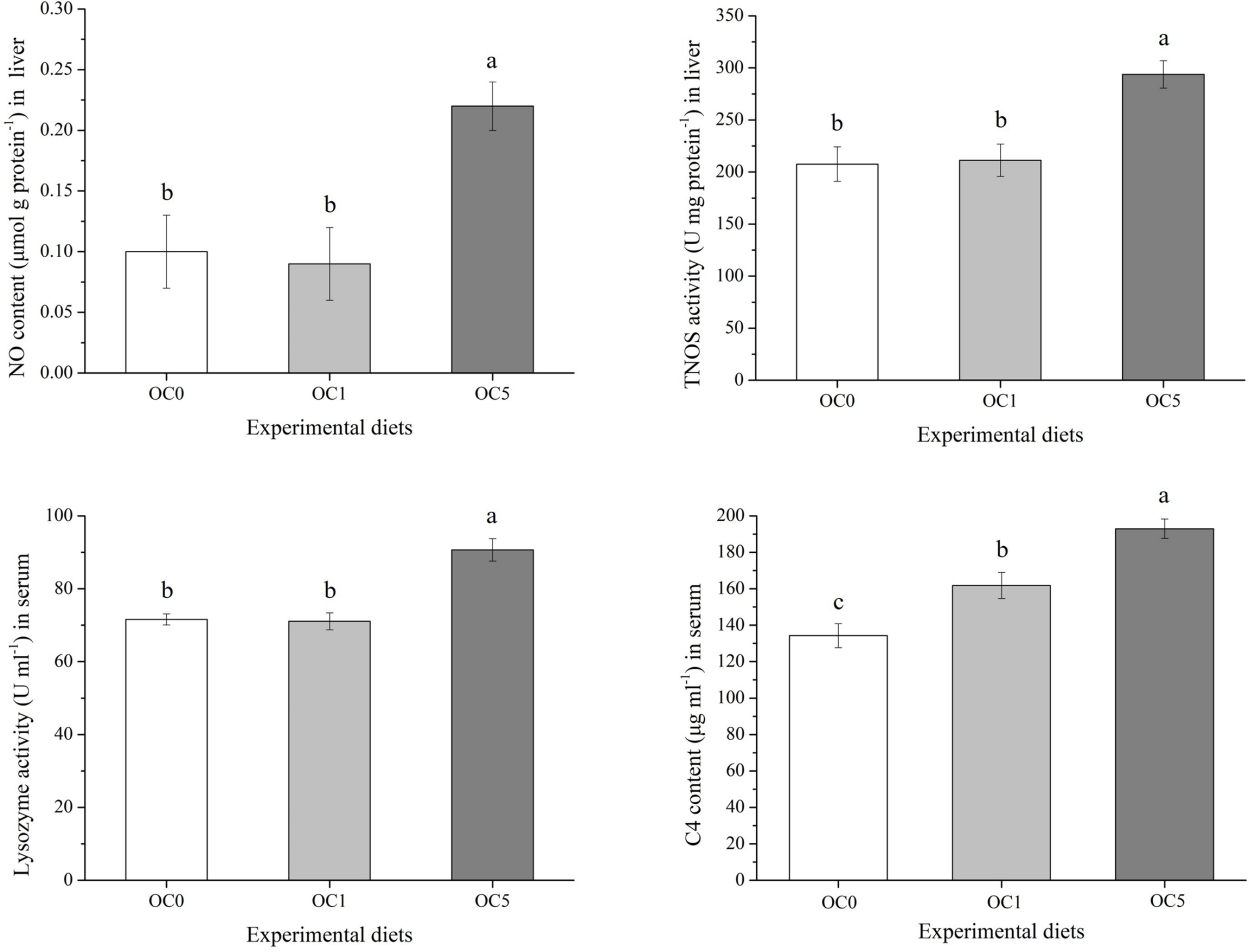
Immune-related parameters in the liver and serum of *T. ovatus* fed experimental diets. Values were presented as mean ± SE (n = 3). The small letters indicated significant differences at *P* < 0.05.

As shown in **Figure 4**, the NO content and TNOS activity in the liver as well as lysozyme activity in the serum of the OC5 diet treatment increased significantly compared to the OC0 and OC1 diet treatments (*P* < 0.05). Besides, the C4 content in the serum of the OC1 and OC5 diet treatments increased significantly compared to the OC0 diet treatment.

### Antioxidant-Related, Metabolism-Related, and Immune-Related Parameters

**Figure 5:**
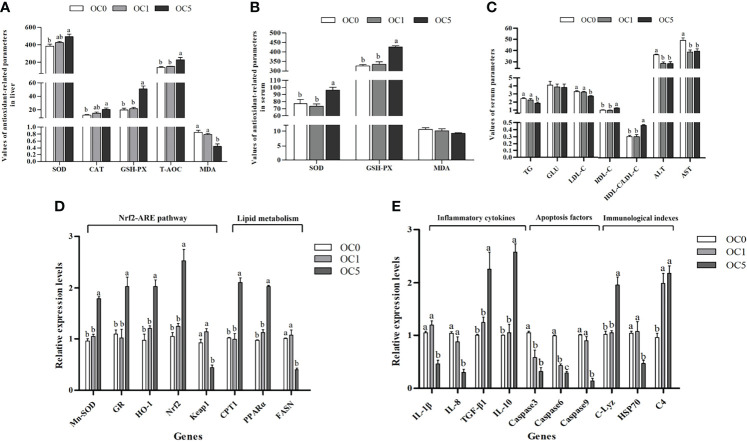
Antioxidant-related parameters in liver **(A)** or serum **(B)**, serum biochemical parameters **(C)**, and relative expression levels of antioxidant-related, metabolism-related, and immune-related genes in the liver **(D, E)** of *T. ovatus* fed experimental diets. SOD, superoxide dismutase (U mg protein^-1^ in liver; U ml^-1^ in serum); CAT, catalase (U mg protein^-1^); GSH-PX, glutathione peroxidase (U mg protein^-1^ in liver; U ml^-1^ in serum); T-AOC, total antioxidant capacity (mmol g protein^-1^); MDA, malondialdehyde (nmol mg protein^-1^ in liver; nmol ml^-1^ in serum); TG, triglyceride (mmol L^-1^); GLU, glucose (mmol L^-1^); ALT, alanine aminotransferase (U L^-1^); AST, aspartate aminotransferase (U L^-1^); LDL-C, low-density lipoprotein cholesterol (mmol L^-1^); HDL-C, high-density lipoprotein cholesterol (mmol L^-1^). Mn-SOD, manganese superoxide dismutase; GR, glutathione reductase; HO-1, hemeoxygenase-1; Nrf2, NF-E2-related nuclear factor 2; Keap1, Kelch-like-ECH-associated protein 1; C-Lyz, c-type lysozyme; HSP70, heat shock protein 70; C4, complement 4; IL-1β, interleukin 1β; IL-8, interleukin 8; TGF-β1, transforming growth factor β1; IL-10, interleukin 10; CPT1, carnitine palmitoyl transferase 1; PPARα, peroxisome proliferator-activated receptors-alpha; FASN, fatty acid synthesis. Values were presented as mean ± SE (n = 3). The small letters indicated significant differences at *P* < 0.05.

As shown in **Figure 5A**, the T-AOC and CAT, GSH-PX, and SOD activities in the liver of the OC5 diet treatment were increased compared with the OC0 diet treatment (*P* < 0.05). Conversely, the MDA content of liver in the OC5 diet treatment was significantly lower than that in the OC0 and OC1 diet treatments (*P* < 0.05). Serum GSH-PX and SOD activities in the OC5 diet treatment were significantly higher than those in the OC0 and OC1 diet treatments (*P* < 0.05) (**Figure 5B**). There was no significant difference in serum MDA content among all diet treatments (*P* > 0.05).

The serum TG and LDL-C contents in the OC5 diet treatment were significantly lower than those in the OC0 and OC1 diet treatments (*P* < 0.05). Conversely, the serum HDL-C content and HDL-C/LDL-C ratio in the OC5 diet treatment were significantly higher than those in the OC0 and OC1 diet treatments (*P* < 0.05). Besides, the activities of ALT and AST in the serum were significantly decreased in the *O. carolinianum* supplementation treatments (*P* < 0.05) (**Figure 5C**).

Compared with the OC0 and OC1 diet treatments, the mRNA transcriptional levels of glutathione reductase (*GR*), NF-E2-related nuclear factor 2 (*Nrf2*), superoxide dismutase (*Mn-SOD*), hemoxygenase-1 (*HO-1*), carnitine palmitoyltransferase 1 (*CPT1*), and peroxisome proliferator-activated receptor-alpha (*PPARα*) were significantly enhanced in the OC5 diet treatment (*P* < 0.05). Conversely, fish fed the OC5 diet showed significantly lower mRNA transcriptional levels of Kelch-like-ECH-associated protein 1 (*Keap1*) and fatty acid synthesis (*FASN*) (*P* < 0.05) (**Figure 5D**).

*O. carolinianum* supplementation significantly down-regulated the mRNA transcriptional levels of *caspase 3* and *caspase 6* and up-regulated the mRNA transcriptional level of complement 4 (*C4*) (*P* < 0.05). Compared with the OC0 and OC1 diet treatments, the mRNA transcriptional levels of interleukin 1β (*IL-1β*), interleukin 8 (*IL-8*), *caspase 9*, and heat shock protein 70 (*HSP70*) were significantly reduced in the OC5 diet treatment (*P* < 0.05). Conversely, fish fed the OC5 diet showed significantly higher mRNA transcriptional levels of interleukin 10 (*IL-10*), transforming growth factor β1 (*TGF-β1*), and c-type lysozyme (*C-Lyz*) (*P* < 0.05) (**Figure 5E**).

## Discussion

Astaxanthin could increase nutrient utilization by regulating the intermediate metabolic process and ultimately result in improved growth performance of fish (26). Previous studies have reported that dietary astaxanthin or *H. pluvialis* powder supplementation enhanced the growth performance in *P. crocea* (9), *T. ovatus* (6, 27), and *Astronotus ocellatus* (28). Similar results were found in the present study. *T. ovatus* fed the OC5 diet showed a significantly elevated growth performance (SGR and WGR) and reduced FCR. There is a close correlation between digestive enzyme activity and feed utilization efficiency, which can be used to evaluate the digestive and absorptive capacities of fish (29, 30). For fish, high activities of digestive enzyme can promote the digestion and absorption of nutrients and ultimately improve growth performance (29, 31). Besides, intestinal morphology is also closely related to digestion ability. Long villus with an increased surface area is conducive to enhance the digestion ability of the intestinal tract to nutrients, which has a positive effect on the growth performance of fish (22, 32). In the present study, the activities of LPS and PEP and the length of villus in the midgut were increased in the OC5 diet treatments. Therefore, dietary *O. carolinianum* supplementation enhanced the growth and the feed utilization efficiency of *T. ovatus* may be mainly attributed to the positive effects of microalgal powder supplementation on villus length and digestive enzymes activities in the midgut.

In the current study, liver morphological examination showed pathological alterations in fish fed the OC0 diet, including obvious infiltration of inflammatory cells and extensive lipid droplets, which were all common lipid deposition characteristics. Fish cultured in offshore cages are more vulnerable to various environmental stressors (e.g., typhoons, rainstorm, hypoxia, temperature, pollutants), which may suppress fatty acid β-oxidation and ultimately result in lipid deposition and peroxidation in the liver (22, 33, 34). Besides, previous studies have suggested that hepatocyte apoptosis is an important element of liver damage and is associated with the occurrence and development of inflammatory responses (22, 35, 36). Therefore, to further clarify the hepatoprotective mechanism of *O. carolinianum*, the current study determined the effects of *O. carolinianum* on the mRNA transcriptional levels of lipid metabolism-related, inflammation-related, and apoptosis-related genes.

PPARα is a critical transcription regulator involved in fatty acid transport, lipoprotein hydrolysis, and peroxisomal and mitochondrial β-oxidation (37). CPT1, located outside the mitochondrial membrane, is the mitochondrial fatty acid transporter involved in catalyzing fatty acid β-oxidation in mitochondria (38). Activated PPARα enhances the expression levels of fatty acid oxidation-related genes, such as *CPT1*, lipoprotein lipase (*LPL*), and adipose triglyceride lipase (*ATGL*), thereby suppressing lipid accumulation in the liver and decreasing the lipid level in serum (39). FASN is a key enzyme that catalyzes acetyl coenzyme A and malonyl coenzyme A to synthesize long-chain fatty acids (40). In this study, fish fed the OC diet upregulated the mRNA transcriptional levels of *PPARα* and *CPT1* and downregulated the mRNA transcriptional level of *FASN* in the liver. The results demonstrated that 5% *O. carolinianum* exerted hepatoprotective effects by inhibiting lipid synthesis and promoting lipolysis. Besides, lipid deposition is closely related to the occurrence of inflammatory responses. Activated PPARα negatively regulates pro-inflammatory pathways (37). Similar results were found in the present study. Fish fed the OC5 diet showed a significantly lower mRNA expression of pro-inflammatory cytokine genes (*IL-8* and *IL-1β*) and a higher expression of anti-inflammatory cytokine genes (*IL-10* and *TGF-β1*). *O. carolinianum* has an anti-inflammatory property and potently suppresses inflammatory responses in the liver. Apoptosis is the programmed death of cells following inflammation, which plays an important role in regulating the development of inflammatory response and preventing tissue and organ damage after inflammation response. Therefore, hepatocyte apoptosis was evaluated based on the mRNA expression level of caspase family genes (*Caspase 3*, *Caspase 6*, *Caspase 9*). Caspase activity is a critical indicator for detecting hepatocyte apoptosis of fish (35). Caspases can be divided into upstream initiators (e.g., Caspase 8, Caspase 9, Caspase 10) and downstream effectors (e.g., Caspase 3, Caspase 6, Caspase 7) based on the molecular structure and order in the apoptosis program (36). Caspase 9, an initiator of apoptosis program, triggers a cascade of downstream caspase activation (e.g., Caspase 3, Caspase 6) (41). In the current study, the expression levels of *Caspase 3*, *Caspase 6*, and *Caspase 9* conspicuously increased in the liver of fish fed a diet without *O. carolinianum* supplementation, indicating that the apoptotic signal of hepatocytes in the OC0 diet treatment was activated. The activated apoptosis signal may be attributed to the occurrence of inflammatory response in the OC0 diet treatment. Amplified apoptotic signals help to inhibit the development of inflammation by releasing metabolites with anti-inflammatory effects (42). The current results suggested that the liver in the OC0 diet treatment exhibited inflammatory symptoms and activated apoptosis signals, which may be attributed to environmental stress. Therefore, we believe that adding hepatoprotective ingredients to feed is essential for maintaining the liver health of fish cultured in offshore cages. This also proved from another perspective that dietary *O. carolinianum* supplementation exerted a beneficial effect on liver health. *O. carolinianum* has potential as a therapeutic agent for abnormal lipid metabolism, which exerted hepatoprotective effects by modulating lipid metabolism and inhibiting inflammation.

The Nrf2-ARE pathway plays a significant role in protecting cells from oxidative stress by removing reactive oxidants (43). Under basic conditions, Nrf2 and Keap1 form an Nrf2–Keap1 complex anchored in the cytoplasm (44). Once stimulated by stressors, the Nrf2–Keap1 complex dissociates from the cytoplasm and allows Nrf2 to translocate into the nucleus, where it binds to the antioxidant responsive element (ARE) and transcriptionally activates downstream antioxidant enzyme genes, such as *GR*, *HO-1*, and *Mn-SOD* (43, 44). Previous studies reported that astaxanthin exerts protection against oxidative stress by mediating the Nrf2-ARE signaling pathway (45, 46). In this study, the mRNA transcriptional levels of *Nrf2*, *Mn-SOD*, *HO-1*, and *GR* were significantly upregulated and the level of *Keap1* was downregulated in the liver of fish fed the OC5 diet. Moreover, fish fed the OC5 diet showed the highest activities of antioxidant enzymes in the liver (SOD, CAT, GSH-PX) and serum (SOD, GSH-PX). Similar results showed that dietary *H. pluvialis* or astaxanthin supplementation enhanced the activities of antioxidant enzymes in fish (47, 48). Xie etal. (27) suggested that diet-supplemented *H. pluvialis* or astaxanthin improved the antioxidant capacity of *T. ovatus* by activating the Nrf2-ARE signal pathway. Similarly, our previous study found that a diet supplemented with *H. pluvialis* significantly increased the expression levels of Nrf2-ARE pathway-related genes and activities of the antioxidant enzyme in *T. ovatus* (6). T-AOC is usually used to reflect the total antioxidant capacity of fish (49). MDA is a critical indicator for evaluating the damage degree of cell structure and function and the degree of lipid oxidation (50). In the present study, T-AOC in the liver showed a conspicuous increase in the OC5 diet treatment, whereas MDA content was significantly decreased, indicating that a diet supplemented with 5% *O. carolinianum* improved the antioxidant status of the liver of *T. ovatus*. The current results showed that dietary *O. carolinianum* supplementation has a beneficial effect on the antioxidant capacity of *T. ovatus* by mediating the Nrf2-ARE signal pathway and elevating the activities of antioxidant enzymes, and the above characteristics were dose-dependent.

For fish, hematological parameters are critical indicators for evaluating the physiological and pathological changes, which are frequently used for nutritional status assessment and disease diagnosis (51, 52). In addition, such parameters are considered as key evidence to evaluate whether fish health status changes after feeding with additives under culture conditions (53). Previous studies have reported that the activities of serum AST and ALT are critical biomarkers for assessing liver function, and a higher serum level of these enzymes may reflect liver damage and hepatocyte dysfunction (47, 54). In this study, measurement of the liver enzymatic responses showed that the activities of serum AST and ALT significantly decreased in fish fed the diet supplemented with *O. carolinianum*, which may be related to the potential positive effect of *O.carolinianum* on hepatoprotective functions of *T. ovatus*. Similarly, our previous study found that dietary *H. pluvialis* supplementation reduced the activities of serum AST and ALT and improved the liver morphology of *T. ovatus* (6). In the OC0 diet treatment, higher activities of serum AST and ALT may be mainly attributed to the inflammatory symptoms of the liver based on morphological observation. Furthermore, morphological observations also provided strong evidence for the protection of liver health by adding *O. carolinianum* to the diet. Serum cholesterol and triglyceride levels are closely related to the health status of fish (9). LDL is the major carrier that transports cholesterol from the liver to peripheral tissues, which leads to cholesterol deposition and atherosclerosis, whereas HDL is beneficial for cholesterol clearance by transporting cholesterol from peripheral tissues to the liver for catabolism (55, 56). Therefore, the LDL-C/HDL-C ratio is measured as an indicator of transport capacity of cholesterol (55). In the current study, measurement of serum biochemical parameters showed that fish fed the OC5 diet had lower TG and LDL-C and higher HDL-C and HDL-C/LDL-C than those fed other diets. Similar results showed that dietary astaxanthin or *H. pluvialis* supplementation decreased the levels of TG and cholesterol in the serum of *P. crocea* (9). Zhao etal. (6) reported that a diet supplemented with *H. pluvialis* increased the cholesterol transport capacity of *T. ovatus* by enhancing the serum HDL-C level and HDL-C/LDL-C ratio. Serum biochemical parameters are closely related to liver function. The current results indicated that 5% *O. carolinianum* reduced the serum TG level and promoted the transport capacity of cholesterol, which may be mainly attributed to the hepatoprotective effect of *O. carolinianum*.

Compared with other vertebrates, the non-specific immune system of teleost fish plays a more important role in resisting pathogen invasion and secondary damage (53). In the current study, the mRNA transcriptional levels of *C-Lyz* and *C4* were used to evaluate the effect of diet supplemented with *O. carolinianum* on the non-specific immune response of *T. ovatus*. Complement plays a central role in the non-specific immune response of fish and is responsible for removing cellular debris, apoptotic cells, and foreign invaders (57). Besides, it can bind to specific sites on the surface of phagocytes to promote phagocytosis (58). Lysozyme, directly or indirectly together with the complement system, lyses bacteria by hydrolyzing the β-1,4 glycosidic bond of the peptidoglycan layer of the bacterial cell wall (59, 60). A previous study reported that a diet supplemented with astaxanthin or *H. pluvialis* significantly enhanced the lysozyme activity and total complement content in the serum of *P. crocea* (9). Zhao etal. (6) also demonstrated that *T. ovatus* fed *H. pluvialis* up-regulated the mRNA expression of *C-Lyz* and *C4* in the liver. Consistently, the current results found that *T. ovatus* fed *O. carolinianum* up-regulated the mRNA transcriptional level of *C4* and increased the C4 content in the serum. Besides, the mRNA transcriptional level of *C-Lyz* in the liver and Lyz activity in the serum of the OC5 diet group was also significantly higher than that of the OC0 and OC1 diet groups. These results suggested that *O. carolinianum* could improve the non-specific immune response of *T. ovatus* by activating the lysozyme and complement system, and the above characteristics were dose-dependent. In addition, the mRNA transcriptional level of *HSP70* in the liver was determined in order to confirm the hypothesis that the impairment of liver function in the diet without *O. carolinianum* was caused by environmental stressors. HSP70, a biomarker for assessing stress status, can be activated by various environmental stressors, such as thermal shock, hypoxia, pollutants, and heavy metal (61, 62). In the current study, fish fed the OC5 diet down-regulated the mRNA transcriptional level of *HSP70* in the liver. Previous studies suggested that dietary supplemented with immunostimulants reduced the mRNA expression of *HSP70*, which may be due to enhanced tolerance of fish to environmental stressors after feeding with immunostimulants under culture conditions (5, 63). Further study is required to clarify the potential mechanism by which *O. carolinianum* affects stress tolerance and HSP70 mRNA expression in *T. ovatus*. The current results provide strong evidence that fish cultured in offshore cages are susceptible to environmental stressors that can impair liver function.

NO, as a gaseous signaling molecule, is involved in the regulation of neuronal transmission and anti-inflammatory, antitumor, and antibacterial activities (64). NO can react with free radical superoxide to generate active substances, including nitrogen dioxide, dinitrogen trioxide, and peroxynitrite, which cause severe nitrosation and oxidative stress to bacteria, eventually destroying cell membranes and causing cell dysfunction of bacteria (65). Therefore, NO is considered to be an effective bactericidal agent that kills broad-spectrum bacteria, especially drug-resistant ones (66). NO is produced by the NOS-catalyzed reaction of L-arginine with molecular oxygen (67). Therefore, there was a positive correlation between NO content and NOS activity. NO and NOS are considered to be important antibacterial molecules against pathogen infection in aquatic animals (68, 69). In this study, TNOS activity and NO content increased significantly in the liver of fish fed the OC5 diet. The findings obtained in the current study indicated that 5% *O. carolinianum* powder promoted the defense ability of *T. ovatus* against pathogenic infection.

Fatty acid composition is an important indicator reflecting the nutritional value of fish, which has an impact on the sales price. In the current study, fish fed the OC0 or OC1 diet showed similar fatty acid profiles. However, fish fed the OC5 diet showed higher contents of oleic acid and eicosenoic acid and a lower content of linoleic acid in the whole body than that of fish fed other diets, which may be due to the high oleic acid content of the *O. carolinianum* itself. In addition, fish oil content in the feed formula of the OC5 group was reduced in order to maintain the consistency of the total lipid content between experimental diets, which may be the main reason for the reduction of the linoleic acid content of the whole body in the OC5 group. The aforementioned observation suggested that *O. carolinianum* powder can be used by fish and affect the fatty acid profiles of whole body. Furthermore, an attractive result was found in this study, showing that *O. carolinianum* did not contain EPA and DHA under the culture conditions of this experiment, but the partial replacement of fish oil by *O. carolinianum* powder did not have an adverse effect on the contents of DHA and EPA in the whole body. These results indicated that dietary *O. carolinianum* supplementation may promote the synthesis of endogenous DHA and EPA in *T. ovatus*. It is well known that marine species have a lower capability or inability for long-chain polyunsaturated fatty acid (LC-PUFA) biosynthesis (70). However, previous studies demonstrated that fatty acyl desaturase exhibited Δ4, Δ5, Δ6, and Δ8 activities, which plays an important regulatory role in the biosynthesis pathway of endogenous LC-PUFA in *T. ovatus* (71, 72). Therefore, the results obtained in this study also proved that *T. ovatus* may have the ability for *de novo* synthesis of endogenous LC-PUFA and that dietary *O. carolinianum* supplementation promotes this ability. However, further study is required to clarify the potential mechanism by which *O. carolinianum* affects the biosynthesis pathway of endogenous LC-PUFA in *T. ovatus*. Besides, dietary *O. carolinianum* supplementation promoted the deposition of astaxanthin in the whole body. Astaxanthin is beneficial to human health, such as antioxidant, anti-inflammation, antidiabetic, cardiovascular disease prevention, anticancer, and immune modulation (5). Therefore, *T. ovatus* containing astaxanthin is more attractive to consumers, which also contributes to the increase in sales price.

## Conclusions

In summary, this study indicated that *O. carolinianum* exerted beneficial effects in *T. ovatus*. The diet supplementation of 5% *O. carolinianum* increased growth performance, antioxidant ability, and non-specific immunity and exerted the hepatoprotective effects of *T. ovatus*. Besides, dietary *O. carolinianum* supplementation promoted the deposition of astaxanthin in the whole body. Based on the results of this study, 5% *O. carolinianum* is recommended to be added to the diet to promote the growth, antioxidant capacity, immune response, and flesh quality of *T. ovatus*. Then, it is particularly important to further evaluate the optimal addition level of *O. carolinianum* for its application in the commercial feed of *T. ovatus*.

## Data Availability Statement

The raw data supporting the conclusions of this article will be made available by the authors, without undue reservation.

## Ethics Statement

The animal study was reviewed and approved by the Institution Animal Care and Use Committee, Sun Yat-Sen University.

## Author Contributions

JN, C-WZ, and WZ designed the study. B-YG contributed to the cultivation of microalgae. WZ carried out the rearing work. WZ, XC, Z-QW, RY, and S-HX collected experimental samples. WZ measured experimental parameters, analyzed the results, and wrote the paper. All authors contributed to the article and approved the submitted version.

## Funding

This research was supported by Project of the National Natural Science Foundation of China (31872580, 32172982), Project of the Science and Technology of Guangdong Province (2021B0202050002), and Project of the Science and Technology of Guangdong Province (2019B110209005), and the Guangdong Provincial Special Fund for Morden Agriculture Industry Technology Innovation Teams (2019KJ143).

## Conflict of Interest

The authors declare that the research was conducted in the absence of any commercial or financial relationships that could be construed as a potential conflict of interest.

## Publisher’s Note

All claims expressed in this article are solely those of the authors and do not necessarily represent those of their affiliated organizations, or those of the publisher, the editors and the reviewers. Any product that may be evaluated in this article, or claim that may be made by its manufacturer, is not guaranteed or endorsed by the publisher.

## Supplementary Material

Supplementary Table 1 Sequences of primers used for real-time quantitative PCR.
