# Supplementary material for: Beneficial Changes in Growth Performance, Antioxidant Capacity, Immune Response, Hepatic Health, and Flesh Quality of Trachinotus ovatus Fed With Oedocladium carolinianum
Source: Front Immunol. 2022 Jul 4;13:940929. doi: 10.3389/fimmu.2022.940929 (PMC9289517; doi:10.3389/fimmu.2022.940929)
Supplement: Supplementary Table 1 — Sequences of primers used for real-time quantitative PCR. [file Table_1.docx]

Supplementary Table 1 Sequences of primers used for real-time quantitative PCR

| Gene name | Primer sequence (5′-3′) |
| --- | --- |
| *Mn-SOD* | F-AGCCAGCCTCAGCCAAACT  R-GGCGGTGACATCTCCCTTT |
| *GR* | F-GTGTGTGTGGGCAAGGAGGA  R-AGATGAGGTGGGGTGAATGG |
| *HO-1* | F-AGAAGATTCAGACAGCAGCAGAACAG  R-TCATACAGCGAGCACAGGAGGAG |
| *Nrf2* | F-TTGCCTGGACACAACTGCTGTTAC  R-TCTGTGACGGTGGCAGTGGAC |
| *Keap1* | F-CAGATAGACAGCGTGGTGAAGGC  R-GACAGTGAGACAGGTTGAAGAACTCC |
| *C-Lyz* | F-GGAGTCTGGTGTTTCTGCTCTTTG  R-GGTGGCTCTAGTGTTGTAGTTCG |
| *HSP70* | F-TTGAGGAGGCTGCGCACAGCTTGTG  R-ACGTCCAGCAGCAGCAGGTCCT |
| *C4* | F-TGGAGAAAAAGTTAAAGGGGC  R-CAGGAAGGAAGTATGAGCGAGT |
| *Caspase3* | F-GCTGCTCTACTGCTTCTGCTGATG  R-TGGCTGAGGATTGTGATGTTGCTG |
| *Caspase6* | F-CCTTCAGCCACAGAGTAGCACATG  R-CGAATGAGGTGGTGGTGGATGC |
| *Caspase9* | F-GAATGGCGTCCGTCTGGTCATC  R-GGCAGCACGTCTCAGTTCAGC |
| *IL-1β* | F-CGGACTCGAACGTGGTCACATTC  R-AATATGGAAGGCAACCGTGCTCAG |
| *IL-8* | F-TGCATCACCACGGTGAAAAA  R-GCATCAGGGTCCAGACAAATC |
| *TGF-β1* | F-GAGATACGGAAAAGAGTGGGG  R-TGACAAAGCGGGAAGCAAG |
| *IL-10* | F-CTCCAGACAGAAGACTCCAGCA  R-GGAATCCCTCCACAAAACGAC |
| *CPT1* | F-CTTTAGCCAAGCCCTTCATC  R-CACGGTTACCTGTTCCCTCT |
| *PPARα* | F-AATCTCAGCGTGTCGTCTT  R-GGAAATGCTTCGGATACTTG |
| *FASN* | F-GAAGGAGAGGGGGTGGAGTC  R-GTGTGAAGGTGGAGGGTGTG |
| *β-actin* | F-TACGAGCTGCCTGACGGACA  R-GGCTGTGATCTCCTTCTGC |
